# Public awareness and perception towards COVID-19 in Sub-Saharan African countries during the lockdown

**DOI:** 10.34172/hpp.2022.25

**Published:** 2022-08-20

**Authors:** Bernadine N. Ekpenyong, Emmanuel K Abu, Raymond Langsi, Uchechukwu L Osuagwu, Richard Oloruntoba, Godwin Ovenseri-Ogbomo, Chikasirimobi G. Timothy, Deborah D Charwe, Obinna Nwaeze, Christopher P Goson, Chundung A Miner, Tanko Ishaya, Khathutshelo P Mashige, Kingsley E. Agho

**Affiliations:** ^1^Department of Public Health, Faculty of Allied Medical Sciences, College of Medical Sciences, University of Calabar, Cross River State, Nigeria; ^2^Department of Optometry and Vision Science, School of Allied Health Sciences, College of Health and Allied Sciences, University of Cape Coast, Ghana; ^3^Health Division, University of Bamenda, Bambili, Cameroon; ^4^Translational Health Research Institute (THRI), School of Medicine, Western Sydney University, Campbelltown, NSW 2560, Australia; ^5^African Vision Research Institute, Discipline of Optometry, University of KwaZulu-Natal, Westville Campus, Durban, 3629, South Africa; ^6^Bathurst Rural Clinical School, School of Medicine, Western Sydney University, Bathurst 2795 NSW, Australia; ^7^School of Management and Marketing, Curtin Business School, Curtin University, Kent Street, Bentley, WA,Australia; ^8^Department of Optometry, Centre for Health Sciences, University of the Highlands and Islands, Inverness, IV2 3JH, United Kingdom; ^9^Department of Optometry and Vision Science, Faculty of Health Sciences, Mzuzu University, Mzuzu, Malawi; ^10^Tanzania Food and Nutrition Center, P.O.Box 977 Dar es Salaam, Tanzania; ^11^Vancouver Island Health Authority (VIHA), Vancouver, BC, Canada; ^12^Department of Psychiatry, College of Health Sciences, University of Jos, Jos, Nigeria; ^13^Department of Community Medicine, College of Health Sciences, University of Jos, Jos, Nigeria; ^14^Department of Computer Science, University of Jos, Jos, Nigeria; ^15^School of Health Sciences, Western Sydney University, Campbelltown, NSW 2560, Australia

**Keywords:** Coronavirus, Africa, Pandemic, Awareness, Risk perception, Attitude

## Abstract

**Background:** The coronavirus disease (COVID-19) outbreak has caused a universal health crisis resulting in significant morbidities and mortalities particularly among high-risk groups. This study sought to determine regional factors associated with knowledge and attitude towards COVID-19 mitigation practices and risk perception of contracting the disease in Sub-Saharan African (SSA) countries.

**Methods:** A cross-sectional anonymous online study was conducted among 1970 participants between April and May 2020, during the lockdown in many SSA countries. Recruitment of participants was via WhatsApp, Facebook and emails using authors’ networks. The outcome variables were KAP (knowledge, attitudes and practice) of COVID-19 and analysis of variance (ANOVA) with post hoc test was run to assess the level of KAP by four regions in SSA. Simple and multiple linear regression (MLR) analyses were performed to examine factors associated with the outcome measures in the four SSA regions.

**Results:** Mean knowledge (*P*=0.707) and risk perception (*P*=0.904) scores by four regions in SSA did not differ significantly. However, the mean attitude score was higher among West Africans compared with Southern (*P*=0.019) and Central Africans (*P*=0.003). MLR analysis revealed that among those living in West (adjusted coefficient β=-0.83 95% CI: -1.19, -0.48) and Southern Africa (β=-0.91 95% CI: -1.42, -0.40), having a primary or secondary education was associated with a decrease in knowledge scores while not being worried about COVID-19 decreased risk perception scores across the four SSA regions(West [β=-6.57, 95% CI: -7.53, -5.62], East [β=-6.24: 95% CI: -8.34,-4.15], Central [β=-6.51, 95% CI: -8.70, -4.31], and Southern Africa [β=-6.06: 95% CI: -7.51, -4.60]). Except among Southern Africans, participants who practiced self-isolation had positive attitude towards COVID-19.

**Conclusion:** Future research on health education regarding COVID-19 or a future related pandemic in SSA should target people with lower education, those who do not self-isolate, those living in Southern and Western Africa and not worried about contracting COVID-19.

## Introduction

 Upon the emergence of the coronavirus disease (COVID-19), there has been severe disruptions to both human and economic activities across the world.^1^ Several mitigation measures and guidelines to limit the spread of the virus were put in place by governments.^2-4^ With COVID-19 vaccines being rolled out globally,^5,6^ some of these restrictive practices have been relaxed including the resumption of international travels.^7^ There are also fears that some countries may be confronted with new COVID-19 waves.^8^ Case fatality rate for COVID-19 varies across countries, and is currently less than 3% globally, with Africa having a case fatality rate of 2.31%.^9^

 The low incidence of COVID-19 case severity and mortality in Africa has been attributed to the co-existence of malaria in this region.^10^ A recent systematic review^10^ found a low incidence of COVID-19 in malaria-endemic regions supporting the suggestion that COVID-19 poor prognosis may be prevented by malaria. Although Africa appeared to have been spared by the infection partly due to its relatively young population (more than 60% are under the age of 25), recent increases in numbers of COVID-19 deaths, were the highest rate of increase in all World Health Organization (WHO) regions,^9^ occurring in South Africa, Ethiopia, and in Kenya^9^ heightening concerns already expressed by scientists^11^ in the midst of a weakened health care system.^12^ This calls for increased regional surveillance as the region cannot cope with the extra burden from the pandemic.

 Since the outbreak of the COVID-19 pandemic, scientists, researchers, and health professionals across the globe with varied expertise have carried out surveys on knowledge, attitudes and practice (KAP) amongst the general population.^13-19^ While some studies have focused on knowledge and perceptions of health workers on COVID-19,^14,18,20,21^ others have focused on African countries,^15,16,22,23^ and one study included a limited number of countries (South Africa, Kenya, and Nigeria).^5^ Kaur and Gupta found that awareness of the pandemic was high across the countries studied with 94% of all respondents being aware of the current outbreak, while 34% perceived it as a global infection.^5^

 Previous studies^15,16,22,23^ that have examined COVID-19 in Africa, particularly in Sub-Saharan African (SSA) countries only established some basic concepts about knowledge and perception levels on the pandemic in single countries. In addition, some studies^20,21^ considered only non-health care workers, and their conclusions may not be generalized to the wider SSA population. Understanding the knowledge, attitude and risk perception on a wider regional scale is important in guiding government policies geared towards reinforcing COVID-19 preventive measures. It also encourages best practices amongst the general SSA population as well as amongst healthcare workers. This study also investigated lifestyle modifications as a result of the pandemic. The findings of this study will help bridge the research gap from previous studies by including seven African countries representing the four regions of Africa, south of the Sahara.

## Materials and Methods

 A cross-sectional survey was carried out during the lockdown period using a Survey monkey in seven African countries with reported COVID-19 cases. The study population consisted of SSAs who were 18 years and older. The seven countries included Nigeria, Ghana, Cameroon, Kenya, Tanzania, Uganda, and South Africa. An e-link to a self-administered online survey was disseminated via emails, Facebook and WhatsApp, which were frequently used by the residents within the participating countries. As noted previously,^24-26^ online surveys can be administered at a lower cost and higher speed than other forms of interviews, they are more interactive, visual, flexible and do not require that interviewers be present. In addition, people who are busy and would systematically disregard partaking in telephone surveys are willing to answer questions when posted on their computer screens.^27^ This was considered the best option to obtain this important information during the lockdown period, where face to face interview was not possible. Participants were allowed a one-month period to complete the survey. Participation was completely voluntary and there were no special incentives or inducements made available to participants by the researchers. Participation was open to only Africans of age 18 years and older, living in or outside of Africa.

###  Dependent or outcome variables

 This validated self-administered online questionnaire survey tool was initially developed and utilized for similar COVID-19 studies in the past.^14-16,28^ The questionnaire was based on the World Health Organization guidelines for clinical and community management of COVID-19.^29^ Participants were tested using 58 items categorized into: socio-demographics, knowledge, attitude towards COVID-19 preventive practices and risk perception sections. Details of the survey are described elsewhere.^16^ The survey was pilot-tested among few people who did not participate in the final survey. Appropriate modification and additional questions were added based on the results of the pilot study. The outcome variables in this study were KAP of COVID-19 among SSA respondents, and the items are described below

 Knowledge about COVID-19 virus was assessed by 12 items, most of which required a ‘yes (scored as 1)’ or ‘no (scored as 0)’ response and the maximum score was 12 points. Attitude towards the preventive practices put in place during the pandemic was assessed by 11 items including “whether they have gone to any crowded place including religious events?” “If they wore a mask when leaving home?”, and “if in recent days, they have maintained good hand washing hygiene using hand sanitizers or washed their hands with soap for at least 20 seconds each time”. Each question used a Likert scale with five levels with scores ranging from 0 (lowest) to 4 (highest) and the maximum score being 24 points. The risk perception of COVID-19 was tested using 16 items in a Likert scale with five levels. Each item score ranged from 0 (lowest) to 4 (highest) and the maximum score was 20 points. The variables included questions on, how they felt about the quarantine, whether participants think they were at risk of becoming infected, at risk of dying from the infection.

 The Cronbach’s alpha coefficients for the knowledge, risk perception and attitude towards the preventive practice scales were 0.78, 0.74, and 0.73, respectively indicating that the internal consistency of each scale was satisfactory.

###  Independent variables

 The independent variables included the socio-demographics of the participants such as age (categorized as 18-28, 29-38, 29-48 and 49+ years based on distribution), region of origin (West, East, Southern and Central Africa), religion (Christian and others), educational (Postgraduate degree [masters and PhD], Bachelor/undergraduate University degree, primary/secondary school), marital (married/de facto and not married [widowed, divorced, separated, and single]), employment, occupational status (working in healthcare and non-healthcare sectors) and household factors (how many people lived together and whether they lived alone or not).

 Questions on knowledge, perceived risk of infection and attitude towards COVID-19 preventive practices were included when each variable was not listed as the dependent variable in the analysis (see Supplementary file for the items).

###  Statistical analysis

 Analyses were performed on survey data using Stataversion 14.1 (StataCorp. College Station, Texas, USA). Descriptive statistics were used to summarize continuous data including the number of observations used in the calculation (*n*), mean, standard deviation (SD). Categorical data were presented as counts and percentages of each category. Preliminary analysis revealed that the mean and median were similar, and the skewness and kurtosis were close to zero and hence, a one-way analysis of variance (ANOVA) was used to establish whether there were any statistically significant differences between the means of KAP scores by region and followed by pairwise comparisons using Tukey’s post hoc test. For each region, simple linear regression model was run to assess the unadjusted Coefficients. All confounding variables with a *P* value < 0.20 were retained and used to build a multiple linear regression (MLR) model. A manual stepwise backwards model was performed to assess the adjusted estimates for the independent variables and to predict the factors associated with scores of KAP towards COVID-19. Breusch-Pagan test was usedto check the homogeneity of variance (homoskedasticity**)** and multicollinearity using variance inflation factors (VIFs) and the VIF < 4 was considered appropriate.^30^ A *P* value < 0.05 was considered statistically significant.

## Results

###  Characteristics of the sample population 

Table 1 presents the demographic characteristics of the respondents. There were 1970 respondents including 1062 (55%) males who participated in the study. About fifty-six percent (n = 1108) of the respondents were from West Africa and more than 2/3rd had completed university education.

**Table 1:** Descriptive statistics of the respondents’ demographics in Sub-Sahara Africa (SSA)

**Variables**	**West Africa**	**East Africa**	**Central Africa**	**Southern Africa**
No. (%)	1108 (56.2)	210 (10.7)	251 (12.7)	401 (20.4)
Demography				
Age, mean (SD)	34.4 (11.6)	36.2 (11.3)	29.9 (10.5)	34.0 (11.8)
Age category in years	1086 (206)	205	245	391
18-28	402 (37.0)	61 (29.8)	137 (55.9)	165 (42.2)
29-38	300 (27.6)	65 (31.7)	51 (20.8)	101 (25.8)
39-48	254 (23.4)	42 (20.5)	41 (16.7)	74 (18.9)
49+	130 (12.0)	37 (18.1)	16 (6.5)	51 (13.0)
Gender	1089	207	244	390
Males	632 (58.0)	123 (59.4)	110 (45.1)	197 (50.5)
Females	457 (42.0)	84 (40.6)	134 (54.9)	193 (49.5)
Marital status	1092		245	391
Married	504 (46.2)	105 (51.0)	74 (30.2)	158 (40.4)
Not married	588 (53.9)	101 (49.0)	171 (69.8)	233 (59.6)
Highest level of education	1095	205	245	391
Postgraduate degree (Masters /PhD)	373 (34.0)	58 (28.3)	70 (28.6)	119 (30.4)
Bachelor’s degree	497 (45.4)	107 (52.0)	112 (45.7)	189 (48.3)
Secondary/Primary	255 (20.6)	40 (19.5)	63 (25.7)	83 (21.2)
Employment status	1095	205	245	393
Employed	740 (67.6)	139 (67.8)	132 (53.9)	258 (65.6)
Unemployed	355 (32.4)	66 (32.2)	113 (46.1)	135 (34.4)
Religion	1093	206	243	392
Christianity	952 (87.1)	186 (90.3)	215 (88.5)	356 (90.8)
Others	141 (12.9)	20 (9.7)	28 (11.5)	36 (9.2)
Occupation	1068	202	239	363
Non-health care sector	595 (55.7)	141 (69.8)	102 (42.7)	172 (47.4)
Health care sector	473 (44.3)	61 (30.2)	137 (57.3)	191 (52.6)
Do you live alone during COVID-19?	1092	206	244	392
No	891 (81.6)	169 (82.0)	195 (79.9)	317 (80.9)
Yes	201 (18.4)	37 (18.0)	49 (20.1)	75 (19.1)
Number living together	867	209	248	397
< 3 people	280 (32.3)	63 (30.1)	51 (20.6)	102 (25.7)
4-6 people	427 (49.2)	109 (52.2)	120 (48.4)	233 (58.7)
6+ people	160 (18.5)	37 (17.7)	77 (31.0)	62 (15.6)
Are you currently or have you been in self-isolation because of COVID-19?	982	182	221	363
No	680 (69.3)	128 (70.3)	154 (69.7)	238 (65.6)
Yes	302 (30.8)	54 (29.7)	67 (30.3)	125 (34.4)
Have been home quarantined due to Covid-19	979	181	221	364
No	599 (61.2)	110 (60.8)	143 (64.7)	213 (58.5)
Yes	380 (38.8)	71 (39.2)	78 (35.3)	151 (41.5)
How much worried are you about COVID-19	1108	210	251	401
Very worried	301 (27.2)	64 (30.5)	71 (28.3)	124 (30.9)
Somehow worried	394 (35.6)	57 (27.1)	76 (30.3)	127 (31.7)
Not at all	413 (37.3)	89 (42.4)	104 (41.4)	150 (37.4)
How do you feel about the self-isolation?
*Anxious*	870	173	193	299
No	373 (42.9)	70 (40.5)	53 (27.5)	136 (43.2)
Yes	497 (57.1)	103 (59.5)	140 (72.5)	179 (56.8)
*Bored*	908	172	195	310
No	243 (26.8)	64 (37.2)	37 (19.0)	117 (37.7)
Yes	665 (73.2)	108 (62.8)	158 (81.0)	193 (62.3)
*Frustrated*	878	172	198	313
No	467 (53.2)	72 (41.9)	81 (40.9)	136 (43.5)
Yes	411 (46.8)	100 (58.1)	117 (59.1)	177 (56.5)
*Angry*	852	166	188	315
No	685 (80.4)	126 (75.9)	119 (63.3)	238 (79.6)
Yes	167 (19.6)	40 (24.1)	69 (36.7)	61 (20.4)

Note: For each variable, number of responses (denominator) were shown.

###  KAP scores of the different SSA regions 

**Figure 1:**
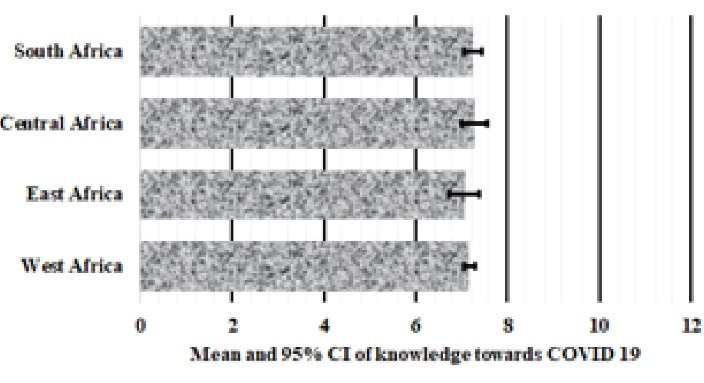
Mean and 95% confidence intervals (CI) of knowledge towards COVID-19 in Sub-Saharan African countries

**Figure 2:**
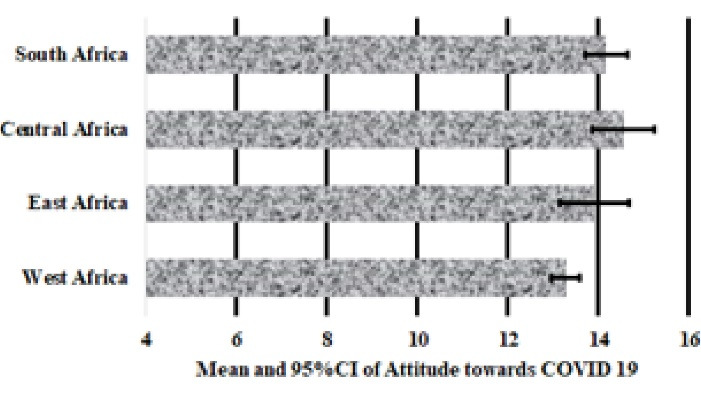
Mean and 95% confidence intervals (CI) of attitude towards COVID-19 in Sub-Saharan African countries

**Figure 3:**
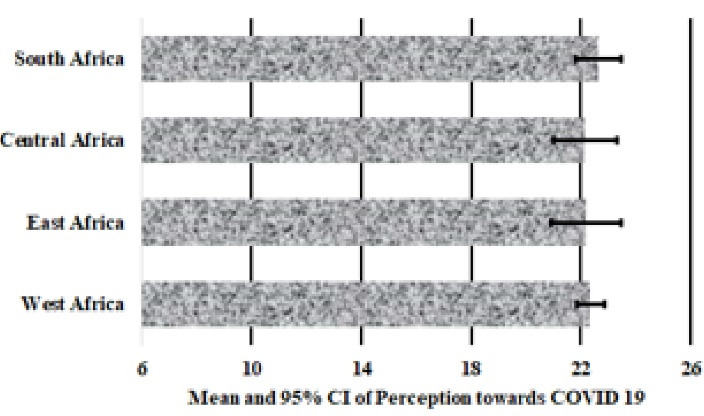
Mean and 95% confidence intervals (CI) of perception towards COVID-19 in Sub-Saharan African countries

Figures 1, 2 and 3 presents the mean and 95% confidence intervals, respectively of knowledge (7.2 ± 0.2), attitude (13.9 ± 0.7) and perception (22.3 ± 0.5) scores of SSA respondents towards COVID-19. A one-way ANOVA found no significant differences in mean scores for knowledge (*P* = 0.707, Figure 1) and perception (*P* = 0.896, Figure 2) between respondents from the Eastern, Western, Central and Southern Africa. However, there was a significant difference in attitude scores between SSA regions (*P* < 0.001) and furthermore, multiple comparison test indicated that West Africans had significantly poorer attitude towards COVID-19 preventive practices compared to Central (*P* = 0.003) and Southern Africans (*P* = 0.019).

###  Factors associated with knowledge of COVID-19 transmission in Sub-Saharan Africa

 The unadjusted and adjusted coefficients of factors associated with COVID-19 related knowledge is presented in Table 2. The findings showed that among respondents from the West, East and Southern Africa, age was associated with COVID-19-related knowledge. Respondents who were aged 29-38 years from West Africa and those aged 49 years and above from East and Southern Africa had significantly higher knowledge about COVID-19 compared to those aged 18–28 years. By contrast, lower knowledge of COVID-19 was observed among Western and Southern African respondents who were single and less educated. Across all SSA regions, respondents that were not worried about contracting the infection showed significantly lower knowledge compared to those who were very worried about contracting the infection. However, after adjustment for confounders, it was revealed that older people living in Central (39-48 years, β = 1.14 95% CI: 0.26, 2.02) and East (49+ years: β = 1.09 95% CI: 0.16, 2.02) Africa and Central Africans with higher education (β = 1.05 95% CI: 0.22, 1.87) were more knowledgeable compared to other respondents (Table 2).

**Table 2:** Unadjusted and adjusted coefficients (95% confidence intervals, CI) of factors associated with knowledge of COVID-19 during the pandemic among Sub-Sahara African respondents

**Variables**	**Unadjusted coefficient (B) (95% CI)**				**Adjusted coefficient (β) (95% CI)**			
	**West Africa**	**East Africa**	**Central Africa**	**Southern Africa**	**West Africa**	**East Africa**	**Central Africa**	**Southern Africa**
Demography								
Age category in years								
18-28	Ref	Ref	Ref	Ref	Ref	Ref	Ref	Ref
29-38	0.49 (0.17, 0.82)	0.61 (-0.19, 1.41)	0.30 (-0.38, 0.99)	0.06 (-0.41, 0.53)	_	0.61 (-0.19, 1.41)	0.78 (-0.01, 1.56)
39-48	0.29 (-0.05, 0.63)	0.53 (-0.37, 1.43)	0.56 (-0.18, 1.31)	-0.05 (-0.56, 0.47)	_	0.53 (-0.37, 1.43)	1.14 (0.26, 2.02)
49+	0.22 (-0.21, 0.65)	1.09 (0.16, 2.02)	0.46 (-0.65, 1.56)	0.63 (0.03, 1.22)	_	1.09 (0.16, 2.02)	1.27 (-0.01, 2.56)
Gender								
Males	Ref	Ref	Ref	Ref	Ref	Ref	Ref	Ref
Females	-0.25 (-0.51, 0.02)	0.11 (-0.54, 0.76)	0.29 (-0.25, 0.83)	0.01 (-0.36, 0.39)	_	_	_	_
Marital status								
Married	Ref	Ref	Ref	Ref	Ref	Ref	Ref	Ref
Not married	-0.36 (-0.62, -0.10)	-0.26 (-0.90, 0.38)	-0.13 (-0.71, 0.45)	-0.13 (-0.52, 0.25)	_	_	_	_
Highest level of Education								
Postgraduate degree (Masters /PhD)	Ref	Ref	Ref	Ref	Ref	Ref	Ref
Bachelor’s degree	-0.24 (-0.53, 0.05)	-0.05 (-0.78, 0.69)	0.3 (-0.34, 0.94)	-0.30 (-0.72, 0.12)	-0.25 (-0.53, 0.05)	_	1.05 (0.22, 1.87)	-0.30 (-0.72, 0.12)
Secondary/Primary	-0.83 (-1.19, -0.48)	-0.32 (-1.25, 0.61)	0.07 (-0.66, 0.80)	-0.91 (-1.42, -0.40)	-0.83 (-1.19, -0.48)	_	0.81 (-0.08, 1.70)	-0.91 (-1.42, -0.40)
Employment status								
Employed	Ref	Ref	Ref	Ref	Ref	Ref	Ref	Ref
Unemployed	-0.26 (-0.54, 0.02)	-0.40 (-1.08, 0.27)	-0.12 (-0.65, 0.42)	-0.31 (-0.71, 0.09)	_	_	_	_
Religion								
Christianity	Ref	Ref	Ref	Ref	Ref	Ref	Ref	Ref
Others	-0.21 (-0.60, 0.17)	-0.45 (-1.53, 0.64)	0.66 (-0.18, 1.50)	0.10 (-0.55, 0.75)	_	_	_	_
Occupation								
Non-health care sector	Ref	Ref	Ref	Ref	Ref	Ref	Ref	Ref
Health care sector	0.07 (-0.23, 0.38)	-0.36 (-1.22, 0.50)	0.02 (-0.70,0.73)	-0.26 (-0.78, 0.26)				
Number living together								
< 3 people	Ref	Ref	Ref	Ref	Ref	Ref	Ref	Ref
4-6 people	-0.15 (-0.49, 0.19)	-0.00 (-0.74, 0.74)	-0.25 (-0.99, 0.48)	-0.16 (-0.63, 0.31)	_	_	_	_
6+ people	0.11 (-0.33, 0.54)	0.35 (-0.62, 1.32)	-0.15 (-0.94,0.65)	-0.14 (-0.79, 0.50)	_	_	_	_
Are you currently or have you been in self-isolation because of COVID-19?
No	Ref	Ref	Ref	Ref	Ref	Ref	Ref	Ref
Yes	0.07 (-0.06, 0.19)	0.07 (-0.17, 0.31)	-0.07 (-0.41, 0.26)	0.01 (-0.17, 0.19)	_	_	_	_
Home quarantined due to Covid-19								
No	Ref	Ref	Ref	Ref	Ref	Ref	Ref	Ref
Yes	0.08 (-0.04, 0.20)	-0.07(-0.29, 0.16)	-0.04 (-0.36, 0.28)	-0.13 (-0.31, 0.04)	_	_	_	_
Do you live alone during COVID-19?								
No	Ref	Ref	Ref	Ref	Ref	Ref	Ref	Ref
Yes	-0.30 (-0.63, 0.03)	-0.63 (-1.44, 0.19)	0.43 (-0.24, 1.10)	0.29 (-0.20, 0.77)	_	_	_	_
How much worried are you about COVID-19?
Very worried	Ref	Ref	Ref	Ref	Ref	Ref	Ref	Ref
Somehow worried	-0.02 (-0.33, 0.29)	-0.03 (-0.84, 0.77)	0.36 (-0.33, 1.05)	0.08 (-0.40, 0.56)	_	_	_	_
Not at all	-1.54 (-1.85, -1.23)	-1.60 (-2.32, -0.87)	-1.32 (-1.97, -0.68)	-1.23 (-1.70, -0.77)	_	_	_	_
How do you feel about the self-isolation?								
*Anxious*								
No	Ref	Ref	Ref	Ref	Ref	Ref	Ref	Ref
Yes	-0.10 (-0.41, 0.20)	-0.08 (-0.79, 0.63)	-0.03 (-1.02,0.41)	-0.04 (-0.52, 0.44)				
*Bored*								
No	Ref	Ref	Ref	Ref	Ref	Ref	Ref	Ref
Yes	-0.06 (-0.39, 0.27)	-0.19 (-0.92, 0.53)	0.31 (-0.51, 1.12)	0.19 (-0.30, 0.68)	_	_	_	_
*Frustrated*								
No	Ref	Ref	Ref	Ref	Ref	Ref	Ref	Ref
Yes	0.03 (-0.27, 0.33)	-0.67 (-1.40, 0.05)	-0.05 (-0.68, 0.58)	0.04 (-0.45,0.53)	_	_	_	_
*Angry*								
No	Ref	Ref	Ref	Ref	Ref	Ref	Ref	Ref
Yes	0.02 (-0.37, 0.40)	-0.49 ( -1.32,0.34)	-0.23 (-0.86, 0.40)	0.37 (-0.24, 0.98)	_	_	_	_

0.00 = Reference; CIs excluding 0.00 are significant variables. For each region, a linear regression model was conducted with knowledge of COVID-19 mean score as the outcome variable, however, only the significant variables after adjusting for potential confounders were presented.

###  Factors associated with attitude towards coronavirus (COVID-19) preventive practices in Sub-Saharan African regions 

Table 3 presents the unadjusted and adjusted coefficients for attitude towards COVID -19 preventive measures during the pandemic. Before adjusting for confounders, positive attitude towards COVID-19 preventive practices during the pandemic was associated with older age such that respondents living in West Africa aged 29-38 years and 49+ years and those aged 29-38 years from East Africa had more positive attitudes towards COVID-19 preventive practices compared to those aged 18-28 years. After adjusting for confounders, positive attitude was significantly associated with the practice of self-isolation while negative attitude was associated with being somewhat worried or not at all worried about getting the infection among Africans except East Africans.

**Table 3:** Unadjusted and adjusted coefficients (95% confidence intervals, CI) of factors associated with attitude towards coronavirus (COVID-19) preventive practices during the pandemic among Sub-Sahara African respondents

**Variables**	**Unadjusted Coefficient B (95% CI)**				**Adjusted Coefficient β (95% CI)**			
	**West Africa**	**East Africa**	**Central Africa**	**Southern Africa**	**West Africa**	**East Africa**	**Central Africa**	**Southern Africa**
Demography								
Age category in years								
18-28	Ref	Ref	Ref	Ref	Ref	Ref	Ref	Ref
29-38	1.59 (0.83, 2.35)	1.99 (0.04, 3.94)	0.65 (-1.08, 2.38)	-0.89 (-2.04, 0.26)	0.64 (0.18, 1.09)	_	_	_
39-48	0.70 (-0.09, 1.50)	0.69 (-1.51, 2.88)	-0.04 (-1.91, 1.84)	0.29 (-.98, 1.56)	0.35 (-0.16, 0.85)	_	_	_
49+	1.17 (0.16,2.17)	1.82 (-0.46, 4.10)	0.97 (-1.81, 3.76)	0.78 (-0.67, 2.24)	0.53 (-0.04, 1.11)	_	_	_
Gender								
Males	Ref	Ref	Ref	Ref	Ref	Ref	Ref	Ref
Females	-0.19 (-0.81, 0.42)	0.19 (-1.38, 1.77)	-0.20 (-1.55, 1.16)	0.34 (-0.58, 1.27)	_	_	_	_
Marital Status								
Married	Ref	Ref	Ref	Ref	Ref	Ref	Ref	Ref
Not married	-0.90 (-1.51,-0.30)	-0.11 (-1.66, 1.44)	0.25 (-1.21, 1.72)	0.33 (-0.61, 1.27)	_	_	_	_
Highest level of Education								
Postgraduate degree (Masters /PhD)	Ref	Ref	Ref	Ref	Ref	Ref	Ref	Ref
Bachelor’s degree	-0.99 (-1.67,-0.31)	-0.11 (-1.91, 1.69)	0.89 (-0.71, 2.49)	-0.08 (-1.13, 0.97)	_	_	_	_
Secondary/Primary	-1.66 (-2.50, -0.82)	-0.45 (-2.71, 1.83)	0.54 (-1.28, 2.37)	-1.55 (-2.83, -0.26)	_	_	_	_
Employment status								
Employed	Ref	Ref	Ref	Ref	Ref	Ref	Ref	Ref
Unemployed	-0.90 (-1.55, -0.26)	-1.10 (-2.74, 0.53)	-0.53 (-1.87, 0.82)	-0.65 (-1.63, 0.33)	-0.60 (-1.04, -0.16)	_	_	_
Religion								
Christianity	Ref	Ref	Ref	Ref	Ref	Ref	Ref	Ref
Others	-0.39 (-1.29, 0.51)	-1.34 (-3.95, 1.28)	1.14 (-0.97, 3.26)	0.76 (-0.83, 2.36)	_	_	_	_
Occupation								
Non-health care sector	Ref	Ref	Ref	Ref	Ref	Ref	Ref	Ref
Health care sector	0.32 (-0.39, 1.03)	-0.63 (-2.68, 1.42)	0.86 (-0.92, 2.65)	0.09 (-1.17, 1.36)	_	_	_	_
Number living together								
< 3 people	Ref	Ref	Ref	Ref	Ref	Ref	Ref	Ref
4-6 people	-0.02 (-0.79, 0.76)	-0.57 (-2.35, 1.21)	-0.29 (-2.11, 1.54)	-0.32 (-1.46, 0.81)	_	_	_	_
6+ people	0.10 (-0.90, 1.10)	0.96 (-1.37, 3.29)	-0.46 (-2.43, 1.51)	-0.57 (-2.11, 0.97)	_	_	_	_
Attitude								
Are you currently or have you been in self-isolation because of COVID-19?
No	Ref	Ref	Ref	Ref	Ref	Ref	Ref	Ref
Yes	1.15 (0.82, 1.48)	1.60 (0.86, 2.33)	1.68 (0.97, 2.40)	0.74 (0.26, 1.23)	0.81 (0.45,1.16)	1.68 (0.91, 2.44)	0.86 (0.04, 1.67)	_
Home quarantined due to Covid-19								
No	Ref	Ref	Ref	Ref	Ref	Ref	Ref	Ref
Yes	1.17 (0.86, 1.47)	1.25 (0.58, 1.92)	1.94 (1.27, 2.61)	0.73 (0.27, 1.20)	1.09 (0.75, 1.43)	_	1.53 (0.75, 2.31)	0.78 (0.32, 1.24)
Do you live alone during COVID-19?
No	Ref	Ref	Ref	Ref	Ref	Ref	Ref	Ref
Yes	-0.30 (-1.08, 0.48)	-1.54( -3.52, 0.45)	0.90 (-0.76, 2.56)	0.59 (-0.59, 1.77)	_	_	_	_
How much worried are you about COVID-19?
Very worried	Ref	Ref	Ref	Ref	Ref	Ref	Ref	Ref
Somehow worried	-0.77 (-1.49,-0.04)	-0.06 (-1.99, 1.86)	-0.73 (-2.44, 0.98)	-1.33 (-2.47, -0.19)	-0.51 (-0.86, -0.15)	_	-0.73 (-2.44, 0.98)	-1.33 (-2.47, -0.20)
Not at all	-3.93 (-4.65,-3.21)	-4.02 (-5.75, -2.28)	-4.19 (-5.78, -2.60)	-3.76 (-4.85, -2.66)	-0.19 (-0.56, 0.19)	_	-4.2 (-5.78,-2.60)	-3.76 (-4.85, -2.66)
How do you feel about the self-isolation?
*Anxious*								
No	Ref	Ref	Ref	Ref	Ref	Ref	Ref	Ref
Yes	-0.00 (-0.71, 0.70)	0.13 (-1.58, 1.85)	-1.07 (-2.86, 0.73)	-0.69 (-1.82, 0.44)	_	_	_	_
*Bored*								
No	Ref	Ref	Ref	Ref	Ref	Ref	Ref	Ref
Yes	0.24 (-0.52, 1.01)	-0.43 (-2.18, 1.32)	-1.15 (-3.18, 0.87)	0.09 (-1.03, 1.22)	_	_	_	_
*Frustrated*								
No	Ref	Ref	Ref	Ref	Ref	Ref	Ref	Ref
Yes	0.14 (-0.56, 0.83)	-1.73 (-3.48, 0.01)	-0.61 (-2.17, 0.97)	-0.51 (-1.63, 0.61)	_	0.71 (0.02, 1.40)	_	_
*Angry*								
No	Ref	Ref	Ref	Ref	Ref	Ref	Ref	Ref
Yes	0.23 (-0.66, 1.12)	-0.87 (-2.89, 1.16)	-1.14 (-2.79, 0.49)	-1.15(-2.80, 0.49)	_	_	_	_
Knowledge	1.62 (1.52, 1.71)	1.80 (1.59, 2.02)	1.65 (1.42, 1.88)	1.65 (1.42, 1.88)	0.35 (0.19, 0.51)	-0.39 (-0.82, 0.05)	0.39 (0.12, 0.66)	0.27 (-0.01, 0.54)

0.00 = Reference; CIs excluding 0.00 are statistically significant variables. For each region, a linear regression model was conducted with mean score for attitude towards preventive practices during the pandemic as the outcome variable. However, only the significant variables after adjusting for potential confounders were presented.

###  Factors associated with perceived risk of contracting COVID-19 in Sub-Saharan African regions 

 The factors associated with respondents’ perceived risk of contracting COVID-19 in SSA are presented in Table 4. The unadjusted results indicated that age differences were associated with the perception of the pandemic in the West and East African sub-regions. Participants within ages 29 -38 years from West and East Africa and those aged 49 years and older from East Africa had significantly higher perception scores compared to those aged 18-28 years. Again, health care sector workers living in West Africa had higher perception than their non-health care sector counterparts (β = 1.09; 95% CI: 0.26, 1.92). On the other hand, lower perception of the infection was significantly linked to lower education and females in West Africa and East Africa respondents who were unhappy for being required to undergo self-quarantine of COVID-19 by their governments.

**Table 4:** Unadjusted and adjusted coefficients (95% confidence intervals, CI) of factors associated with perceived risk of contracting Coronavirus (COVID-19) during the pandemic among Sub-Sahara African respondents

**Variables**	**Unadjusted (B) Coefficient (95% CI)**				**Adjusted (β) Coefficient (95% CI)**			
	**West Africa**	**East Africa**	**Central Africa**	**Southern Africa**	**West Africa**	**East Africa**	**Central Africa**	**Southern Africa**
Demography								
Age category in years								
18-28	Ref	Ref	Ref	Ref	Ref	Ref	Ref	Ref
29-38	1.43 (0.13, 2.74)	3.68 (0.45, 6.90)	2.57 (-0.38, 5.52)	0.16 (-1.84, 2.16)	_	_	_	_
39-48	1.21 (-0.15, 2.58)	1.90 (-1.73, 5.52)	0.46 (-2.74, 3.66)	1.41 (-0.81, 3.62)	_	_	_	_
49+	1.56 (-0.16, 3.29)	4.64 (0.88, 8.41)	0.64 (-4.10, 5.40)	0.93 (-1.61, 3.46)	_	_	_	_
Gender								
Males	Ref	Ref	Ref	Ref	Ref	Ref	Ref	Ref
Females	-1.27 (-2.31, -0.22)	0.61 (-2.00, 3.23)	0.18 (-2.14, 2.50)	-0.04 (-1.65, 1.56)	_	_	_	_
Marital Status								
Married	Ref	Ref	Ref	Ref	Ref	Ref	Ref	Ref
Not married	-0.54 (-1.58, 0.50)	-0.97(-3.54, 1.61)	0.84 (-1.66, 3.35)	0.96 (-2.59, 0.67)	_	_	_	_
Highest level of Education								
Postgraduate degree (Masters /PhD)	Ref	Ref	Ref	Ref	Ref	Ref	Ref	Ref
Bachelor’s degree	-1.28 (-2.45, -0.11)	0.83 (-2.17, 3.82)	0.90 (-1.85, 3.64)	0.68 (-1.15, 2.50)	_	_	_	_
Secondary/Primary	-1.96 (-3.40, -0.52)	-0.08 (-3.85, 3.69)	1.09 (-2.04, 4.21)	-1.50 (-3.73, 0.73)	_	_	_	_
Employment status								
Employed	Ref	Ref	Ref	Ref	Ref	Ref	Ref	Ref
Unemployed	-0.79 (-1.90, 0.31)	-1.81 (-4.54, 0.92)	0.25 (-2.06, 2.55)	-1.41 (-3.10, 0.28)	_	_	_	_
Religion								
Christianity	Ref	Ref	Ref	Ref	Ref	Ref	Ref	Ref
Others	-0.23 (-1.78, 1.31)"	-0.62 (-4.98, 3.73)	2.10 (-1.52, 5.71)	2.21 (-0.55, 4.96)	_	_	_	_
Occupation								
Non-health care sector	Ref	Ref	Ref	Ref	Ref	Ref	Ref	Ref
Health care sector	1.46 (0.26, 2.66)	1.24 (-4.61, 2.13)	0.94 (-2.06, 3.95)	-0.51 (-2.70, 1.69)	1.09 (0.26, 1.92)	_	_	_
Number living together								
< 3 people	Ref	Ref	Ref	Ref	Ref	Ref	Ref	Ref
4-6 people	0.04 (-1.28, 1.35)	0.98 (-1.97, 3.92)	-2.38 (-5.44, 0.69)	-0.61 (-2.57, 1.34)	_	_	_	_
6+ people	0.82 (-0.88, 2.52)	2.46 (-1.40, 6.32)	-3.02 (-6.33, 0.28)	-0.26 (-2.91, 2.39)	_	_	_	_
Are you currently or have you been in self-isolation because of COVID-19?
No	Ref	Ref	Ref	Ref	Ref	Ref	Ref	Ref
Yes	0.22 (-0.55, 0.99)	-0.19 (-2.09, 1.71)	1.22 (-0.72, 3.17)	-0.06 (-1.30, 1.18)	_	_	_	_
Home quarantined due to Covid-19?								
No	Ref	Ref	Ref	Ref	Ref	Ref	Ref	Ref
Yes	0.29 (-0.44, 1.02)	-1.93 (-3.70, -0.17)	1.81 (-0.05, 3.67)	-0.53 (-1.73, 0.66)	_	_	_	_
Do you live alone during COVID-19?								
No	Ref	Ref	Ref	Ref	Ref	Ref	Ref	Ref
Yes	0.49 (-0.85, 1.82)	-1.10 (-4.41, 2.22)	0.95 (-1.92, 3.82)	-0.53 (-2.58, 1.52)	_	_	_	_
How much worried are you about COVID-19?
Very worried	Ref	Ref	Ref	Ref	Ref	Ref	Ref	Ref
Somehow worried	-10.2 (-11.37, -9.05)	-6.64 (-9.65, -3.63)	-5.46 (-8.23, -2.68)	-6.19 (-8.04, -4.34)	-6.34 (-7.27, -5.41)	-6.56 (-8.79, -4.34)	-6.28 (-8.57,-4.00)	-6.37 (-7.84,-4.91)
Not at all	-6.40 (-7.57, -5.23)	-10.27 (-12.98, -7.56)	-9.53 (-12.13, -6.95)	-8.98 (-10.76, -7.21)	-6.57 (-7.53, -5.62)	-6.24 (-8.34, -4.15)	-6.51 (-8.70, -4.31)	-6.06 (-7.51, -4.60)
How do you feel about the self-isolation?
*Anxious*								
No	Ref	Ref	Ref	Ref	Ref	Ref	Ref	Ref
Yes	0.07 (-1.13, 1.27)	0.87 (-1.97, 3.72)	-2.60 (-5.60, 0.40)	-0.83 (-2.82, 1.16)	_	_	_	_
*Bored*								
No	Ref	Ref	Ref	Ref	Ref	Ref	Ref	Ref
Yes	0.40 (-0.89, 1.70)	-1.66 (-4.55, 1.24)	-1.38 (-4.81, 2.06)	0.42 (-1.58, 2.43)	_	_	_	_
*Frustrated*								
No	Ref	Ref	Ref	Ref	Ref	Ref	Ref	Ref
Yes	0.73 (-0.45, 1.91)	-2.26 (-5.18, 0.66)	-1.89 (-4.57, 0.79)	-0.16 ( -2.14, 1.83)	_	_	_	_
*Angry*								
No	Ref	Ref	Ref	Ref	Ref	Ref	Ref	Ref
Yes	-0.24 (-1.75, 1.27)	-3.40 (-6.72, -0.08)	-0.23 (-3.02, 2.56)	-0.01 (-2.48, 2.47)	_	_	_	_
Knowledge	2.57 (2.39, 2.75)	2.75 (2.36, 3.14)	2.46 (2.04, 2.88)	2.57 (2.25, 2.88)	2.35 (2.18, 2.52)	2.53 (2.15, 2.91)	2.29 (1.88, 2.70)	2.38 (2.08, 2.68)

0.00 = Reference; CIs excluding 0.00 are statistically significant variable. For each region, a linear regression model was conducted with mean score for risk perception scores for contracting COVID-19 as the outcome variable. However, only the significant variables after adjusting for potential confounders were presented

 In addition, perceived low risk of contracting COVID-19 was observed amongst individuals living in SSA (Central, East, South and West) who were somehow worried or not worried at all about getting infected with the disease. After correcting for the confounding variables, we found that health care workers and respondents from West Africa, showed high perceived risk of contracting the infection whereas those who were somehow worried or not worried of getting infected had low risk perception of contracting the disease (Table 3). In addition, knowledge of COVID-19 was positively associated with perceived high risk of contracting the infection among SSA respondents.

## Discussion

 This study found that the mean percentage for knowledge, perception and attitude were 58.3%, 28.3% and 54.2%, respectively. This study also revealed that more than half of the respondents in SSA had adequate knowledge about COVID-19 and the preventive measures against it. Older age, higher educational achievement (i.e. bachelor’s degree or more) and being married were associated with high knowledge of COVID-19. However, these were not homogenous across the sub-regions of SSA. Older respondents from Central and Eastern Africa, those from Central Africa who had bachelor’s degree and felt at risk of being infected had good COVID-19 related knowledge while West African respondents who were employed in the health sector had a higher perceived risk of the disease. SSA respondents older than 38 years, those that practiced self-isolation, or self-quarantined in the Central and Southern Africa during the pandemic and knowledgeable West Africans had positive attitude towards COVID-19 preventive practices.

 Our study indicated that three out of every five people surveyed in SSA had good knowledge of COVID-19 which was similar to previous cross-sectional studies conducted in China^14,17,18,31^ which found high knowledge of COVID-19 among the study participants. In this study, two-thirds of the respondents had at least a bachelor’s degree and this may have contributed to the high COVID-19 related knowledge. Although this level of education may not reflect the level of education in the region (UNESCO reported the highest rates of education exclusion in sub-Sahara Africa ),^32^ it is expected that educated people are more inclined to participate in online surveys.^31^ Older people had higher educational qualifications and were more knowledgeable about COVID-19 than the younger age group after adjusting for potential cofounders. This was evident among East and Central African respondents. The COVID-19 pandemic has caused millions of infections and thousands of deaths in Africa and the world in general despite the strict preventive and control public health measures introduced. Similarly, other studies have also reported a positive association between higher educational level and higher knowledge of COVID-19 in the general population^15,19^ and among health care professionals.^21^ The possible reason for this association could be attributed to the fact that people who are more educated are more informed and as such are more likely to update their knowledge of disease using various media. However, some previous studies found significant association between younger age and COVID-19 related knowledge.^14,18^

 In this study, COVID-19 related knowledge was higher among those who stated that they were worried about contracting the infection compared to those who were not worried at all. This is in line with the report that worried individuals were more likely to seek advice or information about a disease during a pandemic.^33^ As the understanding of the epidemiology of COVID-19 evolved, human-to-human transmission was confirmed with the potential for asymptomatic transmission as well.^34^ COVID-19 is transmitted very rapidly such that each patient can spread the virus to two other patients.^35^ This highlights the importance of continuous public education and competency, not only to decrease transmission but to limit anxiety among SSAs, which will result in better compliance, to the mitigation practices put in place by the respective governments. Most of the respondents in this study were worried about contracting the infection (about 60%).

 There was a significant difference in respondents’ attitude towards COVID-19 preventive practices among SSA regions. Although most of the respondents had a generally positive attitude, those from central and southern African countries had greater recognition of the importance of self-quarantine during the pandemic. In the SSA countries where the attitude towards COVID-19 preventive practices were lower (Western and Eastern Africa), there were lower knowledge scores, which was also influenced by their perception of the disease in this study. Other possible barriers against the control measures put in place by governments that could influence attitude include economic factors, poor or non-existent government palliative plan, lack of strict enforcement of the compulsory lockdown, prohibitive cost of face masks and hand sanitizers.^36^

 The perceived risk of contracting the infection was not statistically different across the four SSA regions, but this was significantly influenced by the knowledge of the disease. This explains why perception of the risk of COVID-19 was higher among health workers in West African region but lower among respondents across the four regions who said they were not worried about the COVID-19 disease. The danger based on such insights is that SSA governments might fail to attain the goal of reaching the peak of transmissions and entering the ‘Waning Transmission Phase’ of the pandemic at the end of the lockdown. It is difficult to say if the public health measures are yielding desired results going by the data and other interventions such as the number of testings being undertaken. The exact degree and scale of testing is however, beyond the scope of this study.

 Public health information campaign may also target misinformation on social media as this could affect perception due to misinterpretation of their risk of the health problem. Adjusting these measures without adequate scientific evidence may risk resurgence of COVID-19 cases and jeopardize the efforts of governments and the health of the population. Studies on similar episodes in the past have shown that people’s knowledge, attitude towards COVID-19 preventive practices and perceptions about a condition affects their compliance with public health preventive measures.^33,36-38^

###  Limitations and strengths

 As with most internet-based surveys, the data may be skewed towards using a convenient sample such that only individuals with access to internet and regularly use the social media platforms may have participated in the survey. This may have led to the preponderance of young and educated participants in this study. However, due to the lockdown, this was the only reliable means of disseminating the survey information and online surveys have been shown to have numerous strengths compared to other interview models.^27^ Furthermore, by deploying the questionnaire in the English language only, the study may have excluded the non-English speaking residents in SSA such as the French-speaking people from the Central and West African region. Another limitation of this study was that the lockdown might have limited the participation of respondents, especially in East African countries (Kenya, Uganda and Tanzania) where the citizens were restrained from giving out information regarding the pandemic as was observed by a member of the research team representing those regions. We did not receive assistance with any online company to distribute the survey, which may also have affected the reach of the survey. The results of this study should be interpreted with caution as non-response is not known because we do not know who has received an invitation to participate. In addition, as this was a cross-sectional study and findings may be due chance, the estimates reported may have overestimated or underestimated the level of KAP of COVID-19 in SSA. Despite these limitations, the study has many strengths. Firstly, this is the first study to provide evidence of KAP across different sub-regions of SSA. Secondly, the study tested the hypothesis with robust strategies for controlling confounders at the analysis stage of the research.

## Conclusion

 In conclusion, this study found that the respondents across the four SSA regions have adequate knowledge of COVID-19 with positive attitude towards COVID-19 preventive practices. Future research on health education regarding COVID-19 or future related pandemic in SSA should target people with lower education, those who do not self-isolate, those living in Southern and Western Africa who are not worried about contracting COVID-19. These are important to improve attitude and perceptions of SSAs towards this disease. These findings will help influence decision making by government officials, policy makers and public health workers to direct resources and educational campaigns to target the appropriate personnel.

## Authors’ contributions

 Conceptualization, all authors; methodology, BNE, ULO and KPM; software, KEA and ULO; validation, TI, ON, CPG,RO, BNE, KPM, EKA, ON; CAM and CGT; formal analysis, KEA and ULO; investigation, all authors; resources, all authors; data curation, KEA, CGT, ON, DDC and CPG; writing—original draft preparation, ULO; RL, RL, BNE and EKA; writing—review and editing, TI, RO, BNE, GOO, ULO, KEA, KPM, EKA, CAM, RO and CGT; visualization, CPG and GOO; supervision, KEA, ULO, TI, BNE and KPM; project administration, KEA, ULO and KPM All authors have read and agreed to the published version of the manuscript

## Funding

 The authors did not receive any funding for this study

## Ethical approval

 The study was approved by the Human Research Ethics Committee of the Cross-River State Ministry of Health in Nigeria (Human ethics approval number: CRSMOH/HRP/HREC/2020/117) and adhered to the tenets of the Declaration of Helsinki. Informed consent was also obtained by asking participants to respond either ‘yes’ or ‘no’ to a question asking if they voluntarily wished to participate in the study. Consent was obtained after a detailed explanation of the nature and purpose of the study was provided to all participants in an online preamble. To participate in this study, respondents had to be 18 years and older.

## Competing interests

 The authors declare that they have no conflict of interest.

## Supplementary Files


Supplementary file 1 contains questionnaire.

## References

[1] Guan D, Wang D, Hallegatte S, Davis SJ, Huo J, Li S (2020). Global supply-chain effects of COVID-19 control measures. Nat Hum Behav.

[2] Olshaker M, Osterholm MT (2017). Deadliest Enemy: Our War Against Killer Germs.

[3] Hopman J, Allegranzi B, Mehtar S (2020). Managing COVID-19 in low-and middle-income countries. JAMA.

[4] Yang CJ, Chen TC, Chen YH (2020). The preventive strategies of community hospital in the battle of fighting pandemic COVID-19 in Taiwan. J Microbiol Immunol Infect.

[5] Kaur SP, Gupta V (2020). COVID-19 vaccine: a comprehensive status report. Virus Res.

[6] World Health Organization (who). Evaluation of COVID-19 Vaccine Effectiveness: Interim Guidance, 17 March 2021. WHO; 2021.

[7] Sarwar A, Imran M (2021). Prioritizing infection prevention and control activities for SARS-CoV-2 (COVID-19): a multi-criteria decision-analysis method. J Healthc Leadersh.

[8] ben Khedher N, Kolsi L, Alsaif H (2021). A multi-stage SEIR model to predict the potential of a new COVID-19 wave in KSA after lifting all travel restrictions. Alex Eng J.

[9] Mwai P. Coronavirus: What’s Happening to the Numbers in Africa? BBC News; 2020. Available from: https://africa.cgtn.com/2020/10/18/dr-congos-latest-ebola-outbreak-undercontrol/. Accessed January 14, 2021.

[10] Osei SA, Biney RP, Anning AS, Nortey LN, Ghartey-Kwansah G (2022). Low incidence of COVID-19 case severity and mortality in Africa; could malaria co-infection provide the missing link?. BMC Infect Dis.

[11] Paintsil E (2020). COVID-19 threatens health systems in sub-Saharan Africa: the eye of the crocodile. J Clin Invest.

[12] Nordling L. ‘A ticking time bomb’: scientists worry about coronavirus spread in Africa. Science; 2020. Available from: https://www.science.org/content/article/ticking-time-bomb-scientists-worry-about-coronavirus-spread-africa. Accessed January 14, 2021.

[13] Temsah MH, Alhuzaimi AN, Alamro N, Alrabiaah A, Al-Sohime F, Alhasan K (2020). Knowledge, attitudes and practices of healthcare workers during the early COVID-19 pandemic in a main, academic tertiary care centre in Saudi Arabia. Epidemiol Infect.

[14] Zhong BL, Luo W, Li HM, Zhang QQ, Liu XG, Li WT (2020). Knowledge, attitudes, and practices towards COVID-19 among Chinese residents during the rapid rise period of the COVID-19 outbreak: a quick online cross-sectional survey. Int J Biol Sci.

[15] Abir T, Kalimullah NA, Osuagwu UL, Nur-A Yazdani DM, Mamun AA, Husain T (2020). Factors associated with the perception of risk and knowledge of contracting the SARS-Cov-2 among adults in Bangladesh: analysis of online surveys. Int J Environ Res Public Health.

[16] Ekpenyong BN, Osuagwu UL, Miner CA, Ovenseri-Ogbomo GO, Abu EK, Goson PC (2021). Knowledge, attitudes, and perceptions of COVID-19 among healthcare and non-healthcare workers in sub-Saharan Africa: a web-based survey. Health Secur.

[17] Abdelhafiz AS, Mohammed Z, Ibrahim ME, Ziady HH, Alorabi M, Ayyad M (2020). Knowledge, perceptions, and attitude of Egyptians towards the novel coronavirus disease (COVID-19). J Community Health.

[18] Honarvar B, Lankarani KB, Kharmandar A, Shaygani F, Zahedroozgar M, Rahmanian Haghighi MR (2020). Knowledge, attitudes, risk perceptions, and practices of adults toward COVID-19: a population and field-based study from Iran. Int J Public Health.

[19] Roy D, Tripathy S, Kar SK, Sharma N, Verma SK, Kaushal V (2020). Study of knowledge, attitude, anxiety & perceived mental healthcare need in Indian population during COVID-19 pandemic. Asian J Psychiatr.

[20] Manyaapelo T, Mokhele T, Sifunda S, Ndlovu P, Dukhi N, Sewpaul R (2021). Determinants of confidence in overall knowledge about COVID-19 among healthcare workers in South Africa: results from an online survey. Front Public Health.

[21] Ekpenyong B, Obinwanne CJ, Ovenseri-Ogbomo G, Ahaiwe K, Lewis OO, Echendu DC (2020). Assessment of knowledge, practice and guidelines towards the novel COVID-19 among eye care practitioners in Nigeria-a survey-based study. Int J Environ Res Public Health.

[22] Adesegun OA, Binuyo T, Adeyemi O, Ehioghae O, Rabor DF, Amusan O (2020). The COVID-19 crisis in Sub-Saharan Africa: knowledge, attitudes, and practices of the Nigerian public. Am J Trop Med Hyg.

[23] Workneh F, Wang D, Millogo O, Worku A, Chukwu A, Lankoande B (2021). Knowledge and practice related to COVID-19 and mental health among adults in sub-Saharan Africa. Am J Trop Med Hyg.

[24] Ilieva J, Baron S, Healey NM (2002). Online surveys in marketing research: pros and cons. Int J Mark Res.

[25] Berrens RP, Bohara AK, Jenkins-Smith H, Silva C, Weimer DL (2003). The advent of Internet surveys for political research: a comparison of telephone and Internet samples. Polit Anal.

[26] Manfreda KL, Bosnjak M, Berzelak J, Haas I, Vehovar V (2008). Web surveys versus other survey modes: a meta-analysis comparing response rates. Int J Mark Res.

[27] Kellner P (2004). Can online polls produce accurate findings?. Int J Mark Res.

[28] Ovenseri-Ogbomo GO, Ishaya T, Osuagwu UL, Abu EK, Nwaeze O, Oloruntoba R (2020). Factors associated with the myth about 5G network during COVID-19 pandemic in sub-Saharan Africa. J Glob Health Rep.

[29] World Health Organization (WHO). Survey Tool and Guidance: Rapid, Simple, Flexible Behavioural Insights on COVID-19: 29 July 2020. WHO; 2020.

[30] Vatcheva KP, Lee M, McCormick JB, Rahbar MH (2016). Multicollinearity in regression analyses conducted in epidemiologic studies. Epidemiology (Sunnyvale).

[31] Geldsetzer P (2020). Use of rapid online surveys to assess people’s perceptions during infectious disease outbreaks: a cross-sectional survey on COVID-19. J Med Internet Res.

[32] UNESCO. Education in Africa. UNESCO Institute for Statistics; 2020.

[33] Di Giuseppe G, Abbate R, Albano L, Marinelli P, Angelillo IF (2008). A survey of knowledge, attitudes and practices towards avian influenza in an adult population of Italy. BMC Infect Dis.

[34] Lai CC, Shih TP, Ko WC, Tang HJ, Hsueh PR (2020). Severe acute respiratory syndrome coronavirus 2 (SARS-CoV-2) and coronavirus disease-2019 (COVID-19): the epidemic and the challenges. Int J Antimicrob Agents.

[35] Li Q, Guan X, Wu P, Wang X, Zhou L, Tong Y (2020). Early transmission dynamics in Wuhan, China, of novel coronavirus-infected pneumonia. N Engl J Med.

[36] Lucero-Prisno DE 3rd, Adebisi YA, Lin X (2020). Current efforts and challenges facing responses to 2019-nCoV in Africa. Glob Health Res Policy.

[37] Akan H, Gurol Y, Izbirak G, Ozdatli S, Yilmaz G, Vitrinel A (2010). Knowledge and attitudes of university students toward pandemic influenza: a cross-sectional study from Turkey. BMC Public Health.

[38] Barr M, Raphael B, Taylor M, Stevens G, Jorm L, Giffin M (2008). Pandemic influenza in Australia: using telephone surveys to measure perceptions of threat and willingness to comply. BMC Infect Dis.

