# Supplementary material for: Public awareness and perception towards COVID-19 in Sub-Saharan African countries during the lockdown
Source: Health Promot Perspect. 2022 Aug 20;12(2):200–11. doi: 10.34172/hpp.2022.25 (PMC9508392; doi:10.34172/hpp.2022.25)
Supplement: Supplementary file 1 — contains questionnaire. [file hpp-12-200-s001.pdf]

Ekpenyong et al, **Health Promotion Perspectives,** 2022, 12(2), 200-211
doi: 10.34172/hpp.2022.25
https://hpp.tbzmed.ac.ir

Public awareness and perception towards COVID-19 in Sub-Saharan African countries during the lockdown

Bernadine N. Ekpenyong[1], Emmanuel K Abu-[2], Raymond Langsi[3], Uchechukwu L Osuagwu[4,5,6*], Richard Oloruntoba[7], Godwin Ovenseri-Ogbomo[8], Chikasirimobi G. Timothy[9], Deborah D Charwe[10], Obinna Nwaeze[11], Christopher P Goson[12], Chundung A Miner[13],Tanko Ishaya[14], Khathutshelo P Mashige[5], Kingsley E. Agho[4,5,15]

[1]Department of Public Health, Faculty of Allied Medical Sciences, College of Medical Sciences, University of Calabar, Cross River State, Nigeria
[2]Department of Optometry and Vision Science, School of Allied Health Sciences, College of Health and Allied Sciences, University of Cape Coast, Ghana
[3]Health Division, University of Bamenda, Bambili, Cameroon
[4]Translational Health Research Institute (THRI), School of Medicine, Western Sydney University, Campbelltown, NSW 2560, Australia
[5]African Vision Research Institute, Discipline of Optometry, University of KwaZulu-Natal, Westville Campus, Durban, 3629, South Africa
[6]Bathurst Rural Clinical School, School of Medicine, Western Sydney University, Bathurst 2795 NSW, Australia
[7]School of Management and Marketing, Curtin Business School, Curtin University, Kent Street , Bentley, WA,Australia
[8]Department of Optometry, Centre for Health Sciences, University of the Highlands and Islands, Inverness, IV2 3JH, United Kingdom
[9]Department of Optometry and Vision Science, Faculty of Health Sciences, Mzuzu University, Mzuzu, Malawi
[10]Tanzania Food and Nutrition Center, P.O.Box 977 Dar es Salaam, Tanzania
[11]Vancouver Island Health Authority (VIHA), Vancouver, BC, Canada
[12]Department of Psychiatry, College of Health Sciences, University of Jos, Jos, Nigeria
[13]Department of Community Medicine, College of Health Sciences, University of Jos, Jos, Nigeria
[14]Department of Computer Science, University of Jos, Jos, Nigeria
[15]School of Health Sciences, Western Sydney University, Campbelltown, NSW 2560, Australia

Supplementary data file. Sample of survey tool used in the study

CONSENT
I willingly agree to participate in this survey because I am interested in contributing to the knowledge and perceptions on Coronavirus disease (COVID-19) Pandemic. I understand that there are no forms of payments or reward associated with my participation.

UNDERSTOOD, AGREE AND INTERESTED
NOT UNDERSTOOD, DISAGREE AND NOT-INTERESTED

Country of origin
Country of residence
Province/State/County

Gender
MALE
FEMALE

OTHERS

Age (Years)

Marital Status

SINGLE
MARRIED
SEOARATED/DIVORCED
WIDOW/WIDOWER

Religion

MUSLIM
CHRISTIAN
AFRICAN TRADITIONALIST
OTHERS

Highest level of education

PRIMARY SCHOOL
HIGH/SECONDARY SCHOOL
POLYTHECNIC/DIPLOMA
UNIVERSITY DEGREE (Bachelors/Professional)
POSTGRADUATE DEGREE (Masters/PhD)

Employment Status

SELF EMPLOYED
EMPLOYED
UNEMPLOYED
STUDENT/NON-STUDENT

Occupation
Do you live alone?

YES
NO

If you live with family/friends, how many of you live together?
General KNOWLEDGE of COVID-19 Origin and outbreak
Are you aware of the Coronavirus disease (COVID-19) outbreak?

YES
NO

Are you aware of the origin of the Coronavirus disease (COVID-19) outbreak?

YES
NO

Do you think Coronavirus disease (C0VID-19) outbreak is dangerous?

YES
NO

Do you think Public Health Authorities in your country are doing enough to control the Coronavirus disease (C0VID-19) outbreak?

YES
NO

Do you think Coronavirus disease (COVID-19) has little effect(s) on Blacks than on Whites?

YES
NO
NOT SURE

KNOWLEDGE OF PREVENTION

Do you think Hand Hygiene / Hand cleaning is important in the control of the spread of the Coronavirus disease (COVID-19) outbreak

YES
NO
NOT SURE

Do you think ordinary residents can wear general medical masks to prevent the infection by the COVID-19 virus?

YES
NO
NOT SURE

Do you think Coronavirus disease (COVID-19) is associated with 5G communication?

YES
NO
NOT SURE
Do you think antibiotics can be effective in preventing Coronavirus disease (COVID-19) outbreak?

YES
NO
NOT SURE

If yes to Q22 above, have you purchased an antibiotic in response to COVID-19 disease outbreak?

YES
NO

Do you think there are any specific medicines to treat Coronavirus disease (COVID-19)?

YES
NO
NOT SURE

Do you think there would be a vaccine for preventing Coronavirus disease (COVID-19) outbreak in the next 6 months?

YES
NO
NOT SURE

Do you think Coronavirus disease (COVID-19) was designed to reduce world population?

YES
NO
NOT SURE

Knowledge of symptoms

The main clinical symptoms of Coronavirus disease (COVID-19) are: (Type "YES" or "NO" to the suggested options as applicable)

FEVER
FATIGUE
DRY COUGH
SORE THROAT

Unlike the common cold, stuffy nose, runny nose, and sneezing are less common in persons infected with the COVID-19 virus.

TRUE
FALSE
NOT SURE

There currently is no effective cure for COVID-2019, but early symptomatic and supportive treatment can help most patients recover from the infection

TRUE
FALSE
NOT SURE

It is not necessary for children and young adults to take measures to prevent the infection by the COVID-19 virus.

TRUE
FALSE
NOT SURE

COVID-19 individuals cannot spread the virus to anyone if there's no fever.

TRUE

FALSE
NOT SURE

The COVID-19 virus spreads via respiratory droplets of infected individuals

TRUE
FALSE
NOT SURE

Knowledge of prevention

To prevent getting infected by Coronavirus disease (COVID-19), individuals should avoid going to crowded places such as train stations, religious gatherings, and avoid taking public transportation

TRUE
FALSE
NOT SURE

Isolation and treatment of people who are infected with the Coronavirus disease (COVID-19) virus are effective ways to reduce the spread of the virus. The observation period is usually 14 days

TRUE
FALSE
NOT SURE

Not all persons with COVID-2019 will develop to severe cases. Only those who are elderly, have chronic illnesses, and are obese are more likely to be severe cases.

TRUE
FALSE
NOT SURE

Have you or anyone you know been affected by the Coronavirus disease (COVID-19) in any way(s)?

YES
NO

If Yes to Q36 above, how did the Coronavirus disease (COVID-19) affect you or that person you know? (Type "YES" or "NO" as applicable to the listed effects)

LOST JOB
LOST/CLOSED DOWN BUSINESS
CONTRACTED COVID-19
HOSPITALIZED DUE TO COVID-19
COMPLETELY SEPARATED FROM FAMILY
COMPLETELY STRANDED IN A FOREIGN COUNTRY/AWAY FROM REGULAR HOME/IN A DIFFERENT LOCATION FROM USUAL LOCATION OF RESIDENT
PERCEPTION OF RISK OF INFECTION
Risk of becoming infected.

VERY HIGH

HIGH
LOW
VERY LOW
UNLIKELY

Risk of becoming severely infected

VERY HIGH
HIGH
LOW
VERY LOW
UNLIKELY

Risk of dying from the infection

VERY HIGH
HIGH
LOW
VERY LOW
UNLIKELY

How worried are you because of COVID-19?

A GREAT DEAL
A LOT
A MODERATE AMOUNT
A LITTLE
NONE AT ALL

How do you feel about the self-isolation? (Type "YES" or "NO" to the suggested options as applicable)

WORRIED
BORED
FRUSTRATED
ANGRY
ANXIOUS

I consider the self-isolation as necessary and reasonable

STRONGLY AGREE
AGREE
NEITHER AGREE, NOR DISAGREE
DISAGREE
STRONGLY DISAGREE

Do you think that if you are able to hold your breath for 10 seconds, it's a sign that you don't have COVID-19?

YES
NO
NOT SURE

If you drink hot water, it flushes down the virus

STRONGLY AGREE
AGREE
NEITHER AGREE, NOR DISAGREE
DISAGREE
STRONGLY DISAGREE

WE HAVE TWO OUTCOMES VARIABLES FOR CHLOROQUINE STUDY
Perception and Action

Do you believe that Coronavirus disease (COVID-19) can be cured by taking Chloroquine tablets?

YES
NO
NOT SURE

If yes to Q46 above, have you purchased Chloroquine for the Coronavirus (COVID-19)?

YES
NO

How likely do you think Coronavirus disease (COVID-19) will continue in your country?

VERY LIKELY
LIKELY
NEITHER LIKELY, NOR UNLIKELY
UNLIKELY
VERY UNLIKELY

If Coronavirus disease (COVID-19) continues in your country, how concerned would you be that you or your family would be directly affected?

EXTREMELY CONCERNED
CONCERNED
NEITHER CONCERNED, NOR UNCONCERNED
UNCONCERNED
EXTREMELY UNCONCERNED

PRACTICE REGARDIING COVID-19

In recent days, have you gone to any crowded place including religious events?

ALWAYS
SOMETIMES
RARELY
NOT AT ALL
NOT SURE

In recent days, have you worn a mask when leaving home?

ALWAYS
SOMETIMES
RARELY
NOT AT ALL
NOT SURE

In recent days, have you been washing your hands with soap and running water for at least 20 seconds each time?

ALWAYS
SOMETIMES
RARELY
NOT AT ALL
NOT SURE

Are you currently or have you been in (domestic/home) quarantine because of COVID-19?

YES
NO

Are you currently or have you been in self-isolation because of COVID-19?

YES
NO

Since the government gave the directives on preventing getting infected, have you procured your mask and possibly sanitizer?

YES
NO

Have you travelled outside your home in recent days using the public transport

YES
NO

Are you encouraging others that you come in contact with to observe the basic prevention strategies suggested by the authorities?

YES
NO

How much have you changed the way you live your life because of the possibility of continuing of Coronavirus disease (COVID-19)?

A GREAT DEAL
A LOT
A MODERATE AMOUNT
A LITTLE
NONE AT ALL

THANK YOU FOR TAKING OUR SURVEY
